# Spatial Accessibility to Healthcare Services in Metropolitan Suburbs: The Case of Qingpu, Shanghai

**DOI:** 10.3390/ijerph16020225

**Published:** 2019-01-15

**Authors:** Xiaokun Gu, Lufa Zhang, Siyuan Tao, Boming Xie

**Affiliations:** 1 China Institute for Urban Governance, Shanghai Jiao Tong University, Shanghai 200030, China; guxk1980@sjtu.edu.cn (X.G.); bmxie1226@sjtu.edu.cn (B.X.); 2 School of International and Public Affairs, Shanghai Jiao Tong University, Shanghai 200030, China; 3 Shanghai Tong Ce Planning Design Co., Ltd., Shanghai 200023, China; hykayry@126.com

**Keywords:** spatial accessibility, healthcare services, metropolitan suburb, 2SFCA, urban governance, Shanghai

## Abstract

Spatial accessibility is an important factor for planning healthcare services to maintain a quality life for the metropolitan area. The metropolitan suburb is a special area for its location and rapidly changing population during urbanization. Taking Qingpu district, a suburb of Shanghai as a case, this study evaluated the spatial accessibility to healthcare services of 203 villages and neighborhoods based on the Two-Step Floating Catchment Area (2SFCA) method by ArcGIS software. The result shows that the spatial accessibility in the whole district is quite uneven under lower thresholds, and the spatial differences are beyond the traditional zoning of East Qingpu, New City and West Qingpu. The worst accessibility was mainly distributed at the edges of Jinze, Liantang and Zhujiajiao, while the best accessibility was mainly distributed in the New City and the region close around it. The average value of the spatial accessibility in Qingpu is 2.84, with a reach equal under 90 min threshold by bus index of 2.85, or an under 60 min threshold by self-driving index of 2.70. Secondly, the difference shows a new pattern, that is the spatial accessibility could be affected by both the New City and the Central City. Thirdly, the transportation mode, urbanization, the density of road network and bus lines, as well as the number of doctors in each healthcare service would directly affect the spatial accessibility. Lastly, in order to improve the spatial accessibility in metropolitan suburbs, greater effort is needed in increasing the numbers of bus stations and doctors, especially the areas which are farthest from the New City or the Central City, such as Jinze, and Lian Tang town in Qingpu. We acknowledge that the public transportation is vital to the accessibility to healthcare services. We also emphasize that healthcare services should be planned based on the anticipated future trends of population agglomeration. Our results for Shanghai are applicable to other big cities that are experiencing similar rapid urbanization in China, or other developing countries in Southeast Asia, South Asia, South America and Africa.

## 1. Introduction

Healthcare is one of the most basic necessities needed to maintain a civilized society and normal quality of life for its population [1]. In 1978, the International Conference on Primary Healthcare asserted that health is a human right, and healthcare should be accessible, affordable, and socially relevant [2]. In 2005, the 58th World Health Assembly issued a call on member states for universal health coverage, which means that every citizen can use good quality, promotional, preventative, curative, and rehabilitative health services when required, without experiencing financial hardship [3]. To achieve those goals, equity in access to healthcare is fundamental [4,5] because large inequities in access to healthcare resources and services can exacerbate disparities in health outcomes and quality of life [6,7]. To date, residents’ access to healthcare varies across the world, even in developed urban areas, mainly because of uneven distributions of healthcare providers and consumers (spatial factors). It also varies among population groups because of their different socioeconomic and demographic characteristics (non-spatial factors) [8,9,10,11].

Both spatial and non-spatial accessibility are critical [10]. Spatial accessibility is a measure of potential for healthcare delivery and an important component in evaluating a population’s overall access to healthcare [12] because identifying where the truly underserved populations are located is the essential first step toward any meaningful and effective government intervention programs that can narrow gaps in accessing healthcare and promote overall population health [4,13]. Spatial accessibility further differs in the level of services offered to different population groups, which are effected by non-spatial factors [13]. In most developed countries, due to the high urbanization rate, the growth of urban population is very slow. During the past 20 years, the annual growth rate of urban population in high-income countries was less than 1% in 1995–2015 [14]. Then with the excellent health regional planning and superior infrastructure such as road network construction, the spatial accessibility of healthcare has been increasingly improving [15]. However, the urban population in developing countries has increased dramatically in recent years: the annual growth rate was 3.68% in 1995–2015 [14]: in some metropolis, the rate even reached more than 5%. Thus the spatial accessibility of healthcare in those cities, especially the megacities, faces great challenges. On the one hand, the overall accessibility is not good; on the other hand, there is usually a distinct core-edge pattern. The closer to the traditional center, the better the accessibility is, and vice versa [16,17,18]. However, it also shows that the suburbs near downtown areas often face the most severe challenges compared to the far suburbs due to the existence of urban-rural dual structure. The traditional healthcare services are relatively inadequate, but the influx of foreign population has led to a dramatic increase in the entire permanent population [19]. Therefore, to improve the overall accessibility of the city, the suburban area is a key area. However, the characteristics of spatial accessibility of these regions are not as clear as the downtown area. It may fit the large core-edge pattern which means that the core is the downtown of the whole city, the closer to the downtown of the city, the better the accessibility is [20,21,22], or the small core-edge pattern which means that the core is the downtown of the suburb, the closer to the downtown of the suburb, the better the accessibility is [23,24].Furthermore, because of the availability of such regional data, most of the current spatial scales are only on the street level [20,21,22,25,26], if the scale is finer, there should be more accurate results.

Shanghai has the highest urbanization rate in China and is also one of the metropolitans with the highest population densities in the world [27]. The population urbanization in Shanghai has increased dramatically over the past 30 years, reaching 69% in 1993 and 90% in 2015 [28]. In 1978, the population reached 11 million, and then in 2013, it was over 24 million [28]. Shanghai is also undergoing the challenge of improving the spatial healthcare accessibility. In the late 20th century and early 21st century, Shanghai performed standardized transformation of community health services. All the township health centers were transformed into community health centers (CHCs), and county hospitals became district hospitals [29]. In 2009, to enhance the standard of the suburb, Shanghai launched the suburb healthcare institution construction project in which 3 county hospitals were upgraded to a tertiary hospital [30]. In 2011, to improve the accessibility of healthcare services, Shanghai aimed to form a “1560” medical circle, which means that residents can reach the nearest medical institution in 15 min by foot or a tertiary level hospital in 60 min via common public transportation [31].

There are 16 districts in Shanghai. According to the Shanghai Master Plan (1999–2020), they have been divided into three types of areas: Shanghai Proper also known traditionally as the “Central City”, New Cities, and the suburbs. Qinpu district is one of the 16 districts located in western suburb of Shanghai. Moreover, Qingpu district is special and representative because of the following reasons: first, there is a New City located in the central of Qingpu district, which has been completely urbanized. Second, the East Qingpu, which is directly connected to the Central City and the New City, is defined as “rural” but has partly “urban” in lifestyle, industrial structure and landscape. Third, West Qingpu is far from the Central City and is directly connected to the water conservation areas and traditional agricultural areas. It helps West Qingpu maintain its “rural” in landscape, traditional agricultural. In 2000, the population was 0.596 million, there were three county hospitals, 21 township health centers, and 2473 doctors [32]. In 2010, the population increased to 1.081 million, while the doctor’s number just changed slightly to 2682 [33].

Therefore, Qingpu district would be a good case for studying the spatial accessibility of healthcare services in suburb areas during the dramatically urbanization in China or other similar countries. It can help us to answer the following questions:

What is the accurate spatial healthcare accessibility under the small-scale in metropolitan suburb during the rapid urbanization? What factors possibly affect spatial accessibility? Then what kind of policy could help to improve the spatial healthcare accessibility?

By analyzing recent healthcare, population, and road network data released, this paper attempts to provide some insights into these issues. The remainder of the paper is structured as follows: Section 2 describes the study area, data sources, and the Two-Step Floating Catchment Area (2SFCA) method. Section 3 and Section 4 comprise the results and discussion. A brief summary is presented in Section 5.

From a global perspective, hospitals generally provide inpatient and specialist medical services, whereas primary healthcare institutions mainly provide outpatient and general practice services. In China, hospitals are the most important health facilities, providing both outpatient and inpatient care. Therefore, in the manuscript, we used “healthcare” to refer to outpatient services, and both hospitals and primary healthcare institutions are the healthcare services providers.

## 2. Study Area, Data Sources, and Research Methods

### 2.1. Study Area

**Figure 1:**
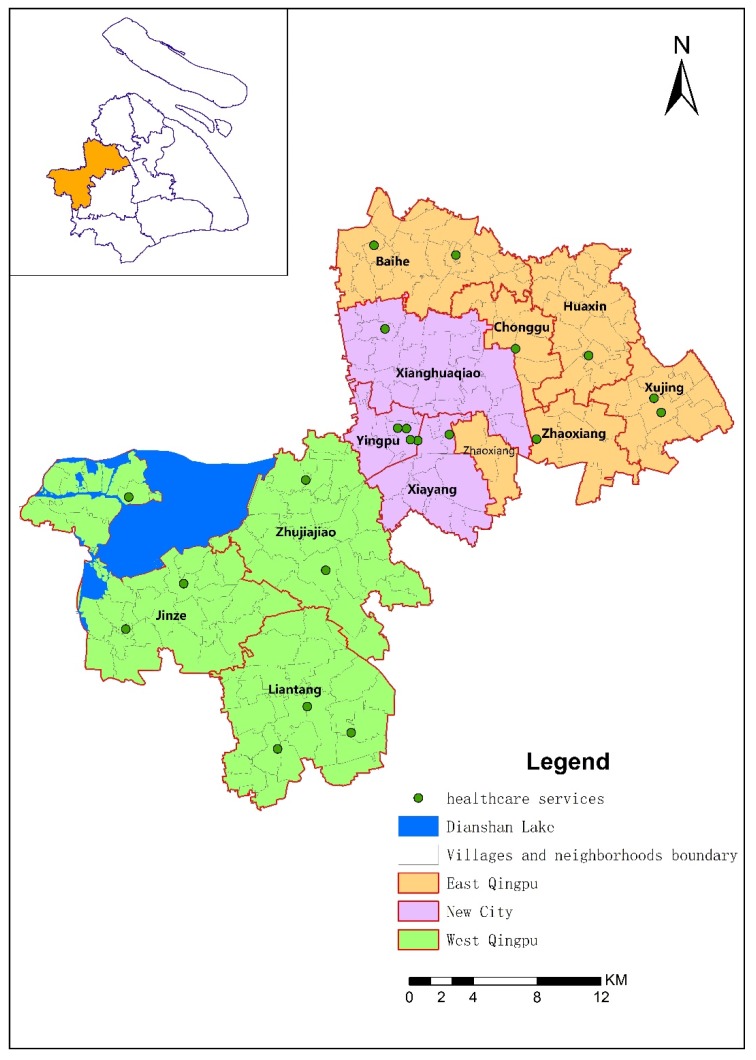
Sub-districts and healthcare services distribution in Qingpu.

Qingpu is located in the western suburbs of Shanghai, 40 km away from the city center landmark named People’s Square (Figure 1).

The total area of Qingpu district is 668.52 km^2^. At the end of 2017, the residential population of Qingpu reached 1.205 million, with a per capita disposable income of 43,225 RMB (around 6294 USD) [34]. The whole district is constituted by 11 subdistrict-level administrative units. As mentioned above, there is a New City located in the central part of Qingpu, thus, the 11 subdistrict-level administrative units could be further divided into two types: those in New City are still called sub-districts, while those in the other areas but New City are called towns. By this definition, Qingpu district has three sub-districts and eight towns. The sub-district includes some neighborhoods, while the town includes a number of villages. In Shanghai, villages and neighborhoods are the smallest public administration spatial unit.

We acknowledge that the division of New City, East Qingpu and West Qingpu exists and is used widely. We adopted this division in our research. As can be seen from the Figure 1, the New City includes Xiayang sub-district, Yingpu sub-district and Xianghuaqiao sub-district. The East Qingpu includes Xujing town, Huaxin town, Baihe town, Zhaoxiang town, Chonggu town. The West Qingpu includes Zhujiajiao town, Liantang town and Jinze town. Figure 1 also shows the distribution of healthcare services: six healthcare services located in the New City, seven healthcare services located in East Qingpu and eight healthcare services located in West Qingpu. Without considering the number of doctors in each healthcare service, the distribution of 21 healthcare services could be seen as relatively balanced in the whole district.

### 2.2. Data Sources

The data mainly included the population and location of villages and neighborhoods, the doctor numbers and location of healthcare services, the road network, as well as the public transportation routes. All data are processed and calculated in ARGGISsoftware (10.2, Environmental Systems Research Institute (ESRI), Redlands, CA, USA).

The population of villages and neighborhoods were obtained from questionnaires because the sub-districts and towns are currently the smallest unit of public statistics provided in China. We investigated each town government and sub-district government for the population in villages and neighborhoods. In total, 164 villages’ and 39 neighborhoods’ populations were retrieved from the local government. We treated them as the 203 evaluation units.

The list of the healthcare services was provided by the health management department of Qingpu district. The numbers of doctors of each healthcare service were retrieved from their official website. A total of 21 healthcare services with 2783 doctors were used to evaluate the spatial accessibility in this paper.

The location of villages and neighborhoods, the location of healthcare services, and the road network were from the ARCGIS data of the 2015 Qingpu land use data and the Qingpu basic geographic information database, which were provided by the Department of Planning and land management of Qingpu. The vectorization of public transport routes is processed by ARCGIS based on the bus line provided by Shanghai Qingpu Bus Public Transport Co., Ltd. (Shanghai, China).

Vehicle speeds on different grades of roads were determined from the corresponding highway technical standard (JTG B01-2014) [35], and bus speeds obtained cited from 2015 *Shanghai Urban Transport Development Report* [36]. These speeds were further discussed with experts from the Qingpu Transportation Commission and then decided for the paper.

### 2.3. Research Methods

As the geographic information system (GIS) was applied to analyze spatial accessibility over 10 years ago, the two-step floating catchment area (2SFCA) method was first proposed by Radke and Mu in 2000 [37] and subsequently revised by Luo and Wang in 2003 [38]. It is widely used in the assessment of spatial accessibility of public service facilities because that it is easy to operate and overcome the limitation of the administrative area boundary of the selected place [11].

#### 2.3.1. Population Distribution

**Figure 2:**
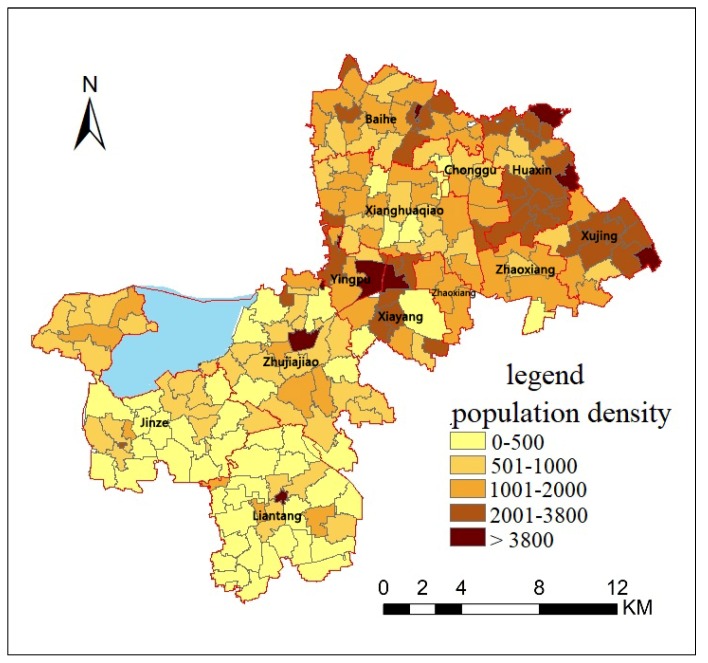
Population density of 203 evaluation units in Qingpu.

The spatial distribution of population density in 203 evaluation units was illustrated in Figure 2. All the units were divided into five levels based on the population density. The higher the level grade, the higher the population density was. The highest population density distributed in Xujing, Huaxin, the center of New City and Zhujiajiao town, with a density of more than 3800 persons per square kilometer. The lowest population density was less than 500 persons per square kilometer, mainly in Jinze and Liantang.

#### 2.3.2. Service Radius and Threshold

In this paper, transportation time was used to express service radius. On the basis of the road conditions in Qingpu and imperfect situation of the suburban rail transit, this study considered the public transportation mode and self-driving mode as the main transportation mode when residents visited health services.

The transportation time, as the service threshold, changed with the transportation mode. Shanghai “1560” strategies prefer to provide the residents healthcare services within 60 min by public transportation. According to the recommendations of six experts in transportation and public administration of Qingpu, we took 30, 45, 60 and 90 min as the thresholds for public transportation. For self-driving, considering that it normally takes less time than public transportation, 20, 30, 40 and 60 min were taken as thresholds.

Experts from the Qingpu Transportation Commission suggested that vehicle speed is affected mainly by two factors: one is the difference of the road grade, the other is the rush hour both in the morning (from 7:00–9:00) and in the evening (from 17:00–19:00). Besides, the driving speed and bus speed were a little different. According to the *2015 Shanghai Urban Transport Development Report* from the Shanghai Institute of Urban and Rural Construction and Transportation Development Research, bus speed during rush hour was about 20–43 km/h, and experts from the Qingpu Transportation Commission suggested that the 30 km/h for an average bus speed would be appropriate, as well as the driving speed by car could be set by the road grade [36]. In this paper, the average bus speed was assumed as 30 km/h (including stopping time at bus stations). The assumed driving speed by car changed with the different road grade: 70, 60, 40, and 30 km/h were assigned to the first-grade roads, elevated and second-grade roads, third-grade road, and fourth-grade road, respectively.

**Figure 3:**
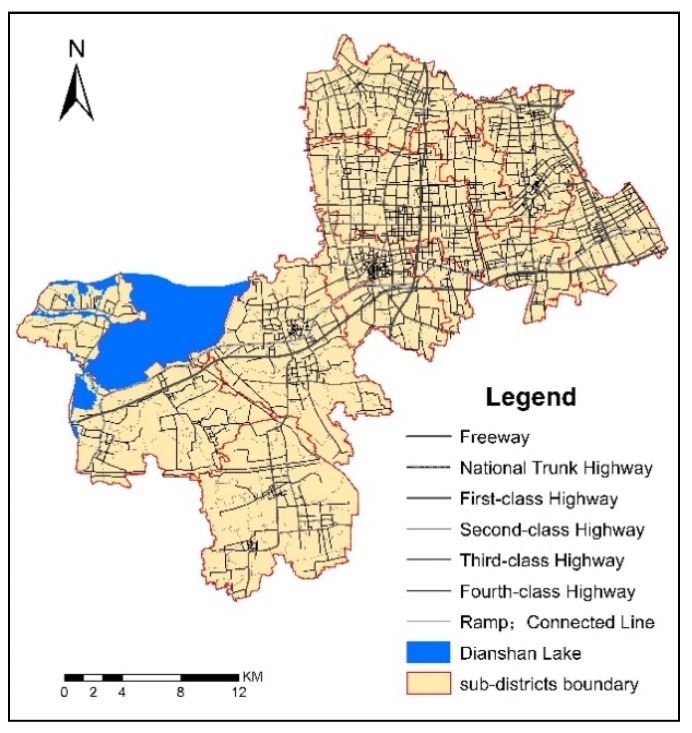
The road network in Qingpu.

**Figure 4:**
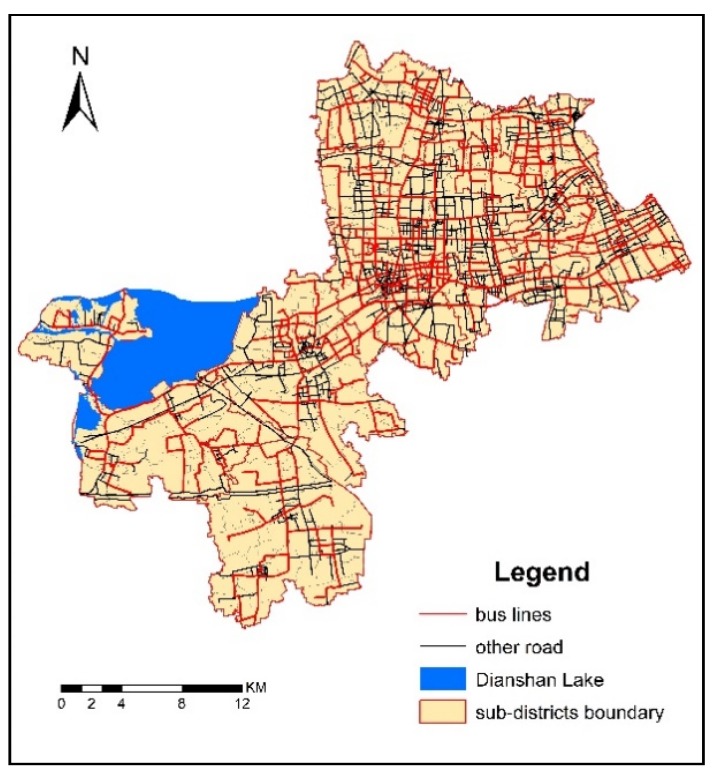
The bus lines in Qingpu.

The service radius (spatial accessibility threshold) of healthcare services was calculated with the network analysis method. The geometric center point was used to represent the healthcare service area. Each healthcare service institution assumes that the service capacity is proportional to its total number of doctors. The road network and bus lines of Qingpu are shown as Figure 3 and Figure 4.

#### 2.3.3. Healthcare Service Spatial Accessibility Index

For each healthcare service point (supply point) *j*, we searched the population of each unit (demand point) *k* within the *j* distance threshold (*d*_0_) (i.e., the search area of *j*) and calculated the supply–demand ratio *R_j_* as follows:

(1) $$R_{j}=\frac{S_{j}}{\sum_{k\in (d_{\mathrm{kj}}\leq d_{0})}D_{k}}$$

where *d_kj_* is the distance between demand point *k* and supply point *j*, *D_k_* is the residents’ demand within the threshold range (i.e., *d_kj_ ≤ d*_0_), and *S_j_* is the total supply of point *j*.

For every homogeneous point (demand point) *i* of units, we searched all healthcare service homogeneous points (supply point) *j* within the threshold range (*d*_0_) (i.e., the search area of *i*). The accessibility of *i* and $A_{i}^{F}$ can be obtained by adding all the supply–demand ratios *R_j_* in the first step as follows:

(2) $$A_{i}^{F}=\sum_{j\in (d_{\mathrm{ij}}\leq d_{0})}R_{j}=\sum_{j\in (d_{\mathrm{ij}}\leq d_{0})}\left[\frac{S_{j}}{\sum_{k\in (d_{\mathrm{ij}}\leq d_{0})}D_{k}}\right]$$

where *d_ij_* is the distance between demand point *i* and supply point *j*, and *R_j_* is the supply–demand ratio of healthcare service *j* in the search area of *i* (*d_ij_* ≤ *d*_0_). Accessibility improves with rising $A_{i}^{F}$.

## 3. Results

Based on the previously presented data and the 2SFCA method, the spatial accessibilities to healthcare services by public transportation and by self-driving in the different threshold were calculated. The higher the accessibility index, the better the healthcare accessibility is to residents living in the village or neighborhood. With the natural breakpoint in ARCGIS, all the accessibility indexes to healthcare services were divided into five grades. The standardization of the accessibility grade to a scale common for all the considered threshold can be shown as Table 1.

For public transportation mode, by bus, with the increase of travel time from 30 min to 90 min, the numbers of evaluation units in each grade centralization had grades of 2.50–3.35. In more details, under 30 min threshold, the difference of spatial accessibility between the evaluation units is higher than that of 45, 60 and 90 min. For self-driving mode, a similar change can be seen in Table 1. With the increase of travel time from 20 min to 60 min, the numbers of evaluation units in each grade centralization had grades of 2.50–3.35.

Because the accessibility indexes of all evaluation units under the thresholds of 90 min by bus and 60 min by self-driving are in the same grade (2.50–3.35), this indicates that under these two thresholds, the healthcare services exhibit a relatively high continuity and the spatial accessibility of all of 203 evaluation units in Qingpu is basically balanced. That is to say, the equality to healthcare services has been achieved under those two thresholds, which will be further analyzed later.

### 3.1. Spatial Accessibility to Healthcare Services by Public Transportation

**Figure 5:**
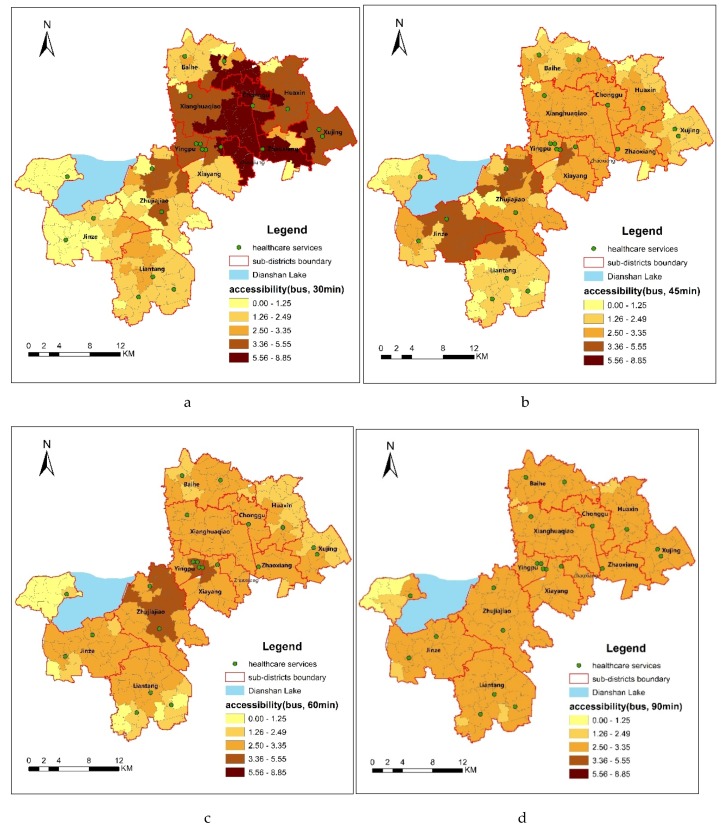
Spatial accessibility by bus under thresholds of (**a**) 30, (**b**) 45, (**c**) 60 and (**d**) 90 min.

The difference of accessibility under 30 min is greater than that under other thresholds by bus. As shown in Figure 5a, the distribution of spatial accessibility of villages and neighborhoods to healthcare services differs significantly under the 30 min threshold. 19.70% of the 203 evaluation units have an index value of 0–1.25, which indicates the worst accessibility. They are mainly distributed throughout Jinze and several villages at the southern tip of Zhujiajiao. 25.21% of the 203 evaluation units have an index value of 1.26–2.49, which indicates poor accessibility. They are mainly distributed throughout Liantang, part of the Baihe town. 15.27% of the 203 evaluation units have an index value of 2.50–3.35, which indicates good accessibility. They are mainly distributed throughout the villages of Liantang town and some neighborhoods of New City. 24.63% of the 203 evaluation units have an index value of 3.36–5.55, which indicates the better accessibility. They are mainly distributed throughout the New City, Huaxin, Xujing, as well as villages of Zhujiajiao that near to the New City. Another 15.27% of the 203 evaluation units have an index value of 5.56–8.85, which indicates the best accessibility. They are mainly distributed throughout the junction between the New City and East Qingpu, including some villages in Huaxin, Xujing, Chonggu, Xianghuaqiao and Baihe. The spatial distribution under 30 min threshold indicates that the good spatial accessibility to healthcare services is beyond the traditional zoning of East Qingpu, New City and West Qingpu.

Under the threshold of 45 min, all the spatial accessibility values are less than 5.56. As shown in Figure 5b, 11.83% of the 203 evaluation units have the better accessibility (3.36–5.55), which mainly distributed in some villages of Jinze and Zhujiajiao that are near to the New City. Meanwhile, 10.84% of the 203 evaluation units which are mainly distributed in those villages of Jinze and Liantang that farthest from the New City have the worst accessibility (0–1.25). Figure 5c shows that the spatial accessibility to healthcare services has become less significant under the 60 min threshold. 65.03% of the 203 evaluation units have an index value of 2.50–3.35. Only 8.38% of the 203 evaluation units, which mainly distributed in the villages of Zhujiajiao, near to the New City, have an index value higher than 3.36. However, there are still 54 (26.61%) evaluation units that have an index value lower than 2.50. They mainly distributed throughout the west of Dianshan Lake of Jinze, and the edge of Liantang, Huaxin and Xujing. Figure 5d shows that 94.09% of the 203 evaluation units have the spatial accessibility of 2.5–3.35 under the thresholds of 90 min by bus, which means that the healthcare services achieved equality for most of the residents in Qingpu.

### 3.2. Spatial Accessibility to Healthcare Services by Self-Driving

**Figure 6:**
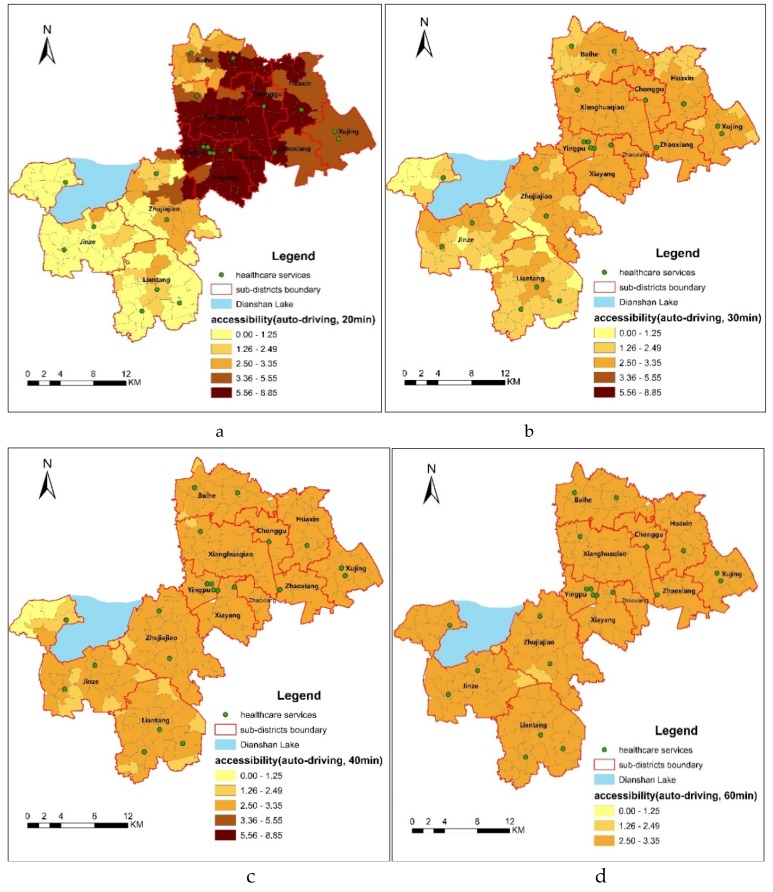
Spatial accessibility by self-driving under thresholds of (**a**) 20, (**b**) 30, (**c**) 40 and (**d**) 60 min.

As shown in Figure 6a, the difference of accessibility under 20 min is greater than that under other thresholds by self-driving. A total of 67 (33.00%) evaluation units have the best accessibility and 57 (28.07%) evaluation units have the worst accessibility. Figure 6a shows where those villages and neighborhoods are located. The best accessibility is mainly distributed throughout the whole New City and Chonggu, and some villages of Huaxin, Baihe and Zhaoxiang. The worst accessibility is mainly distributed throughout most of villages of Jinze and Liantang, as well as villages of Zhujiajiao that are connected with Jinze and Liantang, which are the farthest from the New City and Central City. Besides, 19.21% of the 203 evaluation units have an index value of 3.36–5.55, which indicates better accessibility. They are mainly distributed throughout the whole Xujing, the remaining part of Huaxin, Zhaoxiang and two villages of Zhujiajiao that are close to New City. 10.84% of the 203 evaluation units have an index value of 2.50–3.35, which indicates good accessibility. They are mainly distributed throughout most of Zhujiajiao and some villages of Baihe town and Liantang town. Only 8.87% of the 203 evaluation units have an index value of 1.26–2.49, which indicates poor accessibility.

Under a threshold of 30 min, all the spatial accessibility values are less than 3.35. Figure 6b shows that the spatial accessibility to healthcare services has become less significant under than that under 20 min threshold. 63.55% of the 203 evaluation units have an index value between 2.50–3.35, which is similar to the distribution under 45 min by bus. Fifteen villages (7.30%) which are mainly distributed on the edges of Jinze, Liantang and Zhujiajiao, which are farthest from the Town Center have the worst accessibility, with an index value less than 1.25. Figure 6c shows that the overall distribution of the accessibility to healthcare services was relatively balanced. 86.69% of the 203 evaluation units have an index value of 2.50–3.95, indicating that healthcare service accessibility within the threshold of 40 min was relatively equal in Qingpu. The worst accessibility was distributed to the west of Dianshan Lake of Jinze, and the edge of Liantang. Figure 6d shows that 99.02% of the 203 evaluation units have spatial accessibility between 2.5–3.35 under the thresholds of 60 min by self-driving.

### 3.3. Sensitivity Analysis between the Different Transportation Mode and Travel Time

Using the travel time as service threshold to measure the spatial accessibility under different transportation mode, Table 2 shows a sensitivity analysis of the accessibility to healthcare services when we changed the transportation mode from public transportation to self-driving, as well as the threshold from 20 min to 90 min.

Table 2 shows that the weighted average of the spatial accessibility calculated by two transportation modes and under eight thresholds is 2.84 in the Qingpu district. It also shows that the range of accessibility change from 30 to 45 min is significantly larger than that from 60 to 90 min by bus, as well as the range change from 20 min to 30 min is significantly larger than that from 40 to 60 min by self-driving. This indicates that the smaller the threshold, the greater the change in range of accessibility.

## 4. Discussion

### 4.1. Differences of Spatial Accessibility by Different Transportation Modes

The spatial accessibility to healthcare services in different transportation modes differed. Based on the results (Table 2), the gap between the maximum and minimum values by bus, which indicates the range of accessibility change, is significantly larger than that by self-driving. However, the average of accessibility by bus (index value: 2.84) is slightly lower than that by self-driving (index value: 2.85). This could indicate that a more obvious and sensitive spatial difference of accessibility could be reflected by bus than by self-driving. In general, the self-driving mode accessibility is better than the public transportation mode [21]. This is due to two reasons: first, public transportation routes connecting healthcare institutions and major residential places remain inadequate. Second, the travel time by public transportation is usually longer than the travel time by car because of the stops during the whole trip.

Another point we should pay attention to is that the smaller the threshold, the greater the change in range of accessibility. The results under the thresholds of 30 min by bus and 20 min by self-driving could show a greater difference of accessibility than that under the other thresholds. The spatial accessibility to healthcare services for all residents reached relatively balanced under 60 min, and equal under 90 min by bus; comparatively, the spatial accessibility reached relatively equal under 40 min, and totally balanced under 60 min by self-driving. Furthermore, the average spatial accessibility index by bus changing from 3.36 to 2.51, 2.65 and 2.85 under the different thresholds; similar, the index changing by self-driving from 3.52 to 2.51, 2.67 and 2.70. Therefore, we suggest that the spatial accessibility of healthcare services in Qingpu could basically be equal under 90 min threshold by bus or under 60 min threshold by self-driving. However, when the spatial accessibility is equal, the spatial accessibility by bus (index value: 2.85) is higher than that by self-driving (index value: 2.70). It also indicated that when the threshold becomes larger, the continuity of spatial accessibility increased, while the level of spatial accessibility becomes lower.

Another point we should consider is the travel preferences of residents in Qingpu. We acknowledge that the public transportation is vital to the accessibility to healthcare services. First of all, the public transportation is considered to be more environmental friendly and more sustainable than private vehicles. Many metropolis, including New York, London, Tokyo, and Hong Kong generally encourage public transportation for accessibility to public services. Secondly, self-driving is still a choice for some residents, but quite a few families do not own private cars in Qingpu. According to statistics of Traffic Police Corps of Shanghai Public Security Bureau, the auto owner rate per 100 families was 52% at the end of 2015. For suburban families, this ratio was further reduced. The data of Shanghai’s third agricultural census showed that the auto ownership rate per 100 families in suburban was 40% at the end of 2016. Theoretically, 60% of the families in Qingpu do not have private cars and have to rely on the public transportation for access to healthcare services.

### 4.2. Differences of Spatial Accessibility in the Qingpu

By calculating the spatial accessibility indexes of 203 villages and neighborhoods, this study evaluated the spatial accessibility to healthcare services in Qingpu. The results indicated that the differences of spatial accessibility to healthcare services in the whole district are significant. The most enlightening finding is that the differences of spatial accessibility to healthcare services is beyond the traditional zoning of East Qingpu, New City and West Qingpu, and the results are not similar to those of large scale studies [20,21,22]. Under the threshold of 30 min by bus, the worst accessibility was mainly distributed throughout the Jinze and several villages at the southern tip of Zhujiajiao, while the best accessibility mainly distributed throughout the junction between the New City and East Qingpu, including some villages in Huaxin, Xujing, Chonggu, Xianghuaqiao and Baihe. Under the threshold of 20 min by auto driving, the worst accessibility was mainly distributed throughout most of villages of Jinze and Liantang town, as well as villages of Zhujiajiao that connected with Jinze and Liantang, where they are the most far from the New City and Central City; while the best accessibility was mainly distributed in the whole New City and Chonggu, and some villages of Huaxin, Baihe and Zhaoxiang that are close to New City. The comprehensive spatial accessibility of different traffic modes and thresholds showed that the worst accessibility is mainly distributed in the edges of Jinze, Liatang and Zhujiajiao, which are farthest from the Town Center, and especially the west part of Dianshan Lake; the best accessibility is mainly distributed in the New City and the region close around it, including some villages of Huaxin, Xujing, Chonggu, Xianghuaqiao and Zhujiajiao. Our result indicated another new spatial in metropolitan suburbs, which is the spatial accessibility could be affected by both the New City and the Central City, while the New City affected more than the Central City. It is worth noting that West Qingpu include both the best accessibility in Zhujiajiao and the worst accessibility west of Dianshan Lake.

There are three factors contributing to this difference. The first factor is urbanization. As mentioned above, the New City is a completely urbanized area, while the East Qingpu and West Qingpu are still rural areas. However, East Qingpu is more affected by urbanization than West Qingpu because of its location. Luo and Wang studied the spatial accessibility to health care in Chicago region, including city, suburb and rural, and found a similar spatial variability that better accessibility is in areas closer to the city center [38]. Compared with the Chicago region, the spatial variability in Qingpu is more complex because it is affected by both the Central City and New City. Furthermore, the results indicated that the spatial healthcare accessibility is affected more by the New City than by the Central City. Urbanization contributes greatly to the spatial accessibility of New City and East Qingpu. The second factor is the density of road network and bus lines. As shown in Figure 2 and Figure 3, the density of road network and bus lines in the New City and East Qinpu is obviously higher than that in West Qingpu. In recent years, the population of East Qingpu and the New City has increased rapidly, while the population growth has slowed down or even declined in West Qingpu. Therefore, the infrastructure and public services were mainly concerned in the East Qingpu and the New City. The third factor is the number of doctors in each healthcare service. Although the distribution of 21 healthcare services could be seen as relatively balanced in whole district showed as Figure 1, the number of doctors in each healthcare service is different. The top six hospitals with the largest number of doctors accounted for 48.79% of the total number of doctors, all of them located in the New City. It helps to increase the accessibility indexes by improved the supply capacity of healthcare services in the New City. These three factors could possibly explain the difference of spatial accessibility between the New City, East Qingpu and West Qingpu.

### 4.3. Influences of Planning and Policy

As previously mentioned, Shanghai aimed to form a “1560” medical circle. In another study, we used the Best-Worst Scaling (BWS) method to measure resident’s preferences for seeking healthcare service [39]. Of all the seven attributes, one attribute is the time to reach healthcare institutions by means of mainstream transport. We set three levels on this attribute which are less than 20 min, 30–60 min and more than 60 min. We found the scores of each level were 176, 2 and −172 respectively, which means that residents were very reluctant when the time is over 60 min. Therefore, 60 min is a reasonable threshold when people are seeking for the healthcare service.

In terms of assessing the“1560” medical circle strategy in Qingpu, this paper has not analyzed the residents“15 min on foot” goal yet; However, our research shows that Qingpu has failed to meet the goal of “reaching a tertiary level hospital in 60 min by public transportation” by far. Since the whole district of Qingpu only has one tertiary hospital, namely, Qingpu branch of Zhongshan Hospital Affiliated to Fudan University (Central Hospital of Qingpu District) until 2018, this paper broadened the research objectives to include 21 healthcare services institutions, not only the tertiary level hospital defined by the strategy. The results showed that even by adding all types of healthcare service institutions, it still fails to meet the 60 min spatial accessibility goal. Conversely, in 2018, 50 tertiary hospitals are available in Shanghai, with an average of three hospitals in each district. In addition, over 100 secondary hospitals are functioning in Shanghai, with an average of six hospitals in each district [40]. Thus, the health supply in Qingpu is relatively poor compared with that in Shanghai.

To improve healthcare accessibility in Qingpu, more healthcare service resources should be allocated, especially in West Qingpu. Under the current health practices, the public healthcare service delivery system is still in the leading position. Therefore, the first is the increase of public healthcare service delivery system, including the expansion and construction of community healthcare service institutions and hospitals. In accordance with the number and results of permanent residents, the existing 10 community health service centers should be strengthened through expansion. A new community, with a population of 5–10,000, is planned to be set up with a community health service center or sub-center [41]. In West Qingpu, if we want to reach the theoretical optimum, we should add a hospital among Jinze, Lian Tang and Zhujiajiao towns, but taking the practice and foundation of hospital construction into account, some hospitals such as Zhujiajiao People’s Hospital can be expanded and gradually developed into a high level secondary class comprehensive hospital. At the same time, as an effective complement to the public healthcare system, the government should encourage the development of private healthcare institutions with a certain scale and characteristics in areas where the allocation of healthcare resources is relatively weak.

### 4.4. Potential Limitation and Directions for Further Research

One potential limitation is that the study focuses only on the accessibility to healthcare services of spatial factors but does not consider non-spatial factors. Spatial accessibility would further differ among different population groups if non-spatial factors were considered, which includes the income, education, occupation and such combinations [10]. It is well-documented in many cities and countries that accessibility to public services and healthcare services is often lower in low income groups or lower socio-economic status. Our study has found that spatial accessibility to healthcare services in East Qingpu is higher than that of the New City, while the accessibility of New City is higher than that of West Qingpu. In 2016, the per capita disposable income of residents of East Qingpu, New City and West Qingpu were 33,609–48,998 RMB, 36,616–45,168 RMB and 30,611–32,410 RMB [34]. Further research is needed on the effects of non-spatial factors on accessibility, and it is necessary to include residents’ interviews or investigations to enhance the study.

Another limitation is that the road speed setting. The research reports of traffic management department and the experts’ recommendations were used to estimate the speed of different grades of roads in the study. Big data could be used in the future to make the speed setting more precise. Besides, our study assumed that residents and doctors have no connection with the surrounding areas. However, the healthcare services in the Central City and other districts, as possible service providers, have an impact on residents’ spatial accessibility in Qingpu. Especially with the development of Yangtze River Delta integration, supply and demand for healthcare services will become more complicated.

## 5. Conclusions

By calculating the spatial accessibility indexes to 21 healthcare services based on the 2SFCA method, this study evaluated the spatial accessibility of 203 villages and neighborhoods in Qingpu by public transportation and self-driving in the different threshold. First of all, the weighted average value of the spatial accessibility calculated by two transportation modes and under 8 thresholds is 2.84 in Qingpu district. Secondly, we found that the range of accessibility by bus is larger than by self-driving, as well as the average of accessibility by bus (index value: 2.84) is slightly lower than that by self-driving (index value: 2.85), which could indicate a more obvious and sensitive spatial difference of accessibility could be reflected by bus than by self-driving. Furthermore, the spatial accessibility of healthcare services in Qingpu could basically equal under 90 min threshold by bus or under 60 min threshold by self-driving. However, when the spatial accessibility reached equal, the spatial accessibility by bus (index value: 2.85) is higher than that by self-driving (index value: 2.70).

The results also indicate that the differences of spatial accessibility to healthcare services in whole district are significant. The most enlightening finding is that the differences of spatial accessibility to healthcare services is beyond the traditional zoning of East Qingpu, New City and West Qingpu, and the results are not similar to those of large scale studies. We found that the worst accessibility is mainly distributed on the edges of Jinze, Liantang and Zhujiajiao, which are farthest from the Town center, and especially the west part of Dianshan Lake; the best accessibility is mainly distributed in the New City and the region close around it, including some villages of Huaxin, Xujing, Chonggu, Xianghuaqiao and Zhujiajiao. It is worth noting that West Qingpu includes both the best accessibility in Zhujiajiao and the worst accessibility in west of Dianshan Lake. Besides, the spatial accessibility to healthcare services is most affected in the New City than in the Central City. Our research also shows that even by adding all types of healthcare service institutions, Qingpu still fails to meet the goal of “reaching a tertiary level hospital in 60 min by public transportation” by far. More healthcare service resources should be allocated to Qingpu.

Urbanization, the density of road network and bus lines, as well as the number of doctors in each healthcare service affect directly the spatial accessibility in Qingpu. We acknowledge that the public transportation is vital to the accessibility because 48% of the families in Qingpu do not have private cars, and this rate increased to 60% in West Qingpu. To improve the spatial accessibility in metropolitan suburbs, greater effort is needed in increasing bus stations and the number of doctors, especially the areas which are farthest from the New City or the Central City, such as Jinze, Lian Tang town in Qingpu. We would also like to emphasize that the healthcare services should be planned based on the future trend of population agglomeration caused by important development strategy of Shanghai including the integration of the Yangtze River Delta and the strategy of Rural Revitalization.

## Figures and Tables

**Table 1:** Spatial Accessibility Grade and the numbers of evaluation units for each Grade.

Transportation Mode	Travel Time	Spatial Accessibility Grade				
		0–1.25	1.26–2.49	2.50–3.35	3.36–5.55	5.56–8.85
Bus	30 min	40	51	25	56	31
	45 min	22	58	99	24	0
	60 min	7	47	132	17	0
	90 min	4	8	191	0	0
Self-driving	20 min	57	18	22	39	67
	30 min	15	59	129	0	0
	40 min	4	21	178	0	0
	60 min	0	2	201	0	0

**Table 2:** Sensitivity of accessibility when changing the transportation mode and threshold.

Transportation Mode	Threshold	Minimum Value	Maximum Value	Standard Deviation	Average Value	Weighted Average	Weighted Average
Bus	30 min	0.00	8.86	2.21	3.36	2.84	2.84
	45 min	0.00	5.40	0.98	2.51		
	60 min	0.59	3.65	0.58	2.65		
	90 min	2.33	2.94	0.15	2.85		
Self-driving	20 min	0.00	7.30	2.24	3.52	2.85	
	30 min	0.00	3.34	0.74	2.51		
	40 min	0.80	2.79	0.28	2.67		
	60 min	2.48	2.70	0.00	2.70		
